# Neurocysticercosis in Latin America: Current epidemiological situation based on official statistics from four countries

**DOI:** 10.1371/journal.pntd.0010652

**Published:** 2022-08-29

**Authors:** Roberto Rodríguez-Rivas, Ana Flisser, Luiz Fernando Norcia, Pedro Tadao Hamamoto Filho, D. Katterine Bonilla-Aldana, Alfonso J. Rodriguez-Morales, Arturo Carpio, Matthew L. Romo, Agnès Fleury

**Affiliations:** 1 Instituto Nacional de Neurología y Neurocirugía Manuel Velasco Suarez, Ciudad de México, México; 2 Facultad de Medicina, Universidad Nacional Autónoma de México, Ciudad de México, México; 3 Department of Neurology, Psychology and Psychiatry, Botucatu Medical School. UNESP – Universidade Estadual Paulista, Botucatu, Brazil; 4 Grupo de Investigación Biomedicina, Faculty of Medicine, Fundación Universitaria Autónoma de las Américas, Pereira, Risaralda, Colombia; 5 Master of Clinical Epidemiology and Biostatistics, Universidad Científica del Sur, Lima, Perú; 6 School of Medicine, University of Cuenca, Cuenca, Ecuador; 7 CUNY Institute for Implementation Science in Population Health, City University of New York, New York, United States of America; 8 Departamento de Medicina Genómica y Toxicología Ambiental, Instituto de Investigaciones Biomédicas, Universidad Nacional Autónoma de México, Ciudad de México, México; 9 Clínica de Neurocisticercosis, Instituto Nacional de Neurología Y Neurocirugía Manuel Velasco Suarez, Ciudad de México, México; Institute of Tropical Medicine Antwerp, BELGIUM

## Abstract

**Background:**

Neurocysticercosis (NC) is one of the major parasitic diseases affecting the central nervous system and is endemic in much of Asia, sub-Saharan Africa, and Latin America. Its epidemiology is difficult to assess, although official registries are available in Brazil, Colombia, Ecuador, and Mexico.

**Methodology/Principal findings:**

Using official statistics, we assessed trends in NC hospitalization rates during 1998–2019 in Brazil and Ecuador, during 2004–2019 in Mexico, and during 2009–2019 in Colombia. We also assessed the trend in NC mortality in Brazil (1998–2019), the trend in hospitalizations for NC in a Mexican tertiary-level hospital (Instituto Nacional de Neurología y Neurocirugía [INNN]; 1995–2019), and in Mexican primary care ambulatory clinics (1995–2019). Associations between NC hospitalization rates and the human development index (HDI) were also examined.

In Brazil, Ecuador, and Mexico, statistically significant decreases in NC hospitalization rates were observed. In Mexico, a significant increase in the age of patients at INNN was observed, suggesting a decreasing incidence of recent infection. Conversely, a significant increase in NC hospitalization rate was observed in Colombia. HDI was not significantly associated with NC hospitalization rates when adjusting for time.

**Conclusions:**

The downward trends in NC cases in Brazil, Ecuador, and Mexico are encouraging, especially in the context of the PAHO/WHO plan of action to eliminate neglected tropical diseases from the region. On the other hand, in Colombia, the increased NC hospitalization rate is concerning and needs further evaluation so that the authorities can take specific measures. These results should encourage health authorities in other endemic countries to establish a system of official registries to identify where the need for a control program is most urgent. However, it is also important to remember that NC persists, although less frequently in some Latin American countries, and efforts to achieve its control must continue.

## Author summary

The present work shows the current trends in the frequency of neurocysticercosis hospitalizations in four endemic Latin American countries. Neurocysticercosis is a parasitic disease of the central nervous system that has been historically linked to poverty and poor sanitation. It is one of the neglected tropical diseases included in the PAHO/WHO action plan for their elimination. We compiled official data available from Brazil, Colombia, Ecuador, and Mexico. Our results show a clear and significant reduction in the disease burden in 3 of the 4 countries included. On the other hand, in Colombia, an increase in incidence was observed. These mixed results are of great relevance. They allow us to be optimistic about the possibility of eliminating this disease in some countries, but also allow us to consider why the disease does not seem to be controlled in other countries and direct public health measures aimed at eliminating this parasite in the countries that need it most.

## Introduction

Neurocysticercosis (NC) is a parasitic disease, endemic in most countries in Asia, Sub-Saharan Africa, and Latin America, clearly linked to poverty [1]. Its clinical manifestations are heterogeneous, ranging from headaches, seizures, and focal deficits, although it is estimated that approximately 50% of patients are asymptomatic [2]. Its severity depends mainly on the location of the parasites in the central nervous system [3]. Briefly, the severity is usually lower in the case of parenchymal localisation (symptoms easily controlled by treatment and parasites degenerating rapidly, spontaneously or after cysticidal treatment) than in the case of extraparenchymal localisation (higher severity of symptoms and lesser response to cysticidal treatment) [4]. The diagnosis of NC is based on imaging studies, immunodiagnostic tests, and analysis of the cerebrospinal fluid, which are not widely available or accessible in endemic countries [1].

Partly because of this diagnostic difficulty, data on its current epidemiology and trends in its prevalence and incidence are rarely available in Latin America. Nevertheless, over the last two to three decades, national registries collecting inpatient diagnoses, causes of mortality, and reasons for consultations have been established in several countries. These registries, which use the International Classification of Diseases (ICD) and include data from different health institutions in each country, are a good tool to assess the evolution of the frequency of different diseases over time.

This study aims to examine the trends of NC hospitalization in three Latin American countries *using official national registries* (Brazil, Colombia, and Mexico), and the trend of cysticercosis (not limited to neurological disease) hospitalization in Ecuador. We supplement this information with the NC mortality trend in Brazil and the trends in a tertiary-level hospital and primary care ambulatory clinics in Mexico.

## Methods

Different data of interest were gathered in each participating country. It is important to note that in each of the countries included, reporting to the respective health authorities of inpatient diagnoses in public hospitals, at the secondary and tertiary levels, using ICD-10 (International Classification of Diseases, tenth revision) is mandatory. This is also mandatory for private care in Colombia, Ecuador, and Mexico, but not in Brazil.

### Brazil

Information regarding hospitalization and mortality due to NC between 1998 and 2019 were collected. We used the ICD-10 code B69.0 (Cysticercosis of the Nervous System) to look for patients diagnosed with NC.

For hospitalization data, we used secondary data from DATASUS between 1998 and 2019. These data were extracted through the National Hospitalization Information System (SIH-SUS). This system collects individual-level information (Authorisation of Hospitalization [AIH]) on all hospitalizations occurring in Brazil’s National Health System (SUS—Sistema Único de Saúde, which offers universal coverage for all the Brazilian population and is the only and exclusive option for more than 75% of the population). "Hospitalization" refers to clinical and surgical procedures in qualified hospitals, and each AIH represents the total number of hospitalizations, not the number of patients. In addition, the main diagnosis is defined as a primary cause of hospitalization.

The study included all deaths in Brazil that occurred in the study period regarding mortality, with NC listed on the death certificates as underlying cause of death, similar to the study by Martins-Melo et al., 2016 [5]. The underlying cause of death is the disease that initiated the events leading directly to death.

Hospitalization and mortality rates were calculated, taking into count the Brazilian population reported by the SIH-SUS, and the total number of deaths reported (Sistema de Informações sobre Mortalidade [SIM]; Mortality Information System), in each year of the study.

Official reference population data from the *Instituto Brasileiro de Geografia e Estatística* (IBGE, https://www.ibge.gov.br/en/home-eng.html) was used to estimate annual incidence rates (per 100,000 inhabitants).

### Colombia

We used the same methodology in a published report [6]. Briefly, data were obtained on confirmed NC cases (ICD-10 code B69.0) and were reviewed in terms of quality, initially from data from the Colombian National Institute of Health and afterwards by the Protection Information System (SISPRO) and its Data Cubes system. Given that cysticercosis is not yet under the surveillance system, the epidemiological data were collected from the Individual Health Records System (Registro Individual de Prestación de Servicios, RIPS). Data came from 33 reference notification units, one per department, and were later consolidated and centralised in Bogotá onto the SISPRO system. The Ministry of Health and Social Protection agreed through the Protection Information System (SISPRO) via a client access server: SISPRO RIPS. Official reference population data from the National Administrative Department of Statistics (DANE) was used to estimate annual incidence rates (per 100,000 inhabitants).

### Ecuador

We extracted data from the National Institute of Statistics and Census (INEC), which collects national data from a total of 625 secondary and tertiary institutions including 184 from the public sector and 441 from the private sector. This represents nearly 100% of the total number of Ecuadorian hospitals. Hospitalization rates were calculated, taking into count the total Ecuadorian population and the number of cysticercosis patients (ICD-10 code B.69) in each year of the study (1998–2019). Cysticercosis refers to the presence of larval form of *Taenia solium* in any compartment (such as the central nervous system, muscles, eyes, and subcutaneous tissue). This code does not refer to taeniasis, which is the presence of the adult worm in the intestine.

### Mexico

We gathered the information provided by the General Directorate of Health Information (DGIS) of the Ministry of Health [7], which compiles hospitalization data from all Mexican secondary and tertiary care institutions since 2004. We used the ICD-10 code B.69.0 to look for patients diagnosed with NC and calculated the rate by 100,000 habitants, considering the entire Mexican population (Instituto Nacional de Estadística y Geografía, INEGI, https://www.inegi.org.mx/app/indicadores/).

Data from the National System of Epidemiological Surveillance (SUIVE) between 1995 and 2019, which provides annual information on NC cases reported in primary care ambulatory consultations around the country, was also considered. These rates correspond to the ratio of these data to the total number of ambulatory consultations in Mexican public primary care institutions.

We also updated the frequency of hospitalised patients with NC at the National Institute of Neurology and Neurosurgery Manuel Velasco Suarez (INNN), a tertiary level referral hospital located in Mexico City offering neurological attention to patients from all over the country. We previously published the frequencies of hospitalised patients with NC at the INNN from 1995 to 2009 [8]. In the present study we used the same methodology for collecting data from 2010 to 2019, gathering data from the epidemiology department using the ICD-10 code B.69.0 to look for patients diagnosed with NC using validated diagnostic criteria [9]. The age of the patients was also recorded.

### Human Development Index (HDI)

For all countries, we also gathered the annual human development index (HDI) and assessed its correlation with NC rates. The HDI is a composite statistical index of indicators of life expectancy, education, and per capita income. A country has a higher HDI when these three indicators are higher. Currently (2020 report), Niger has the lowest HDI (0.394) while Norway has the highest (0.957). HDI is used to measure a country’s development by the United Nations Development Programme (UNDP).

### Statistical analysis

Information was analysed with SPSS 25.0 [IBM Corp, Armonk, New York, USA], GraphPad Prism 6.0 for Windows [GraphPad Software, San Diego, California, USA], and SAS 9.4 [SAS Institute, Inc., Cary, North Carolina, USA]. Correlations between NC/CC rates, HDI, age of Mexican patients and calendar periods (in years) and between HDI and NC/CC rates were calculated with the non-parametric Spearman rank correlation test. We used negative binomial regression models for each country, both unadjusted and adjusted for time (calendar year), and offset with log(population), to determine the association of HDI with hospitalization rate. Figures were created using the software GraphPad Prism 6.

## Results

### Brazil

**Fig 1:**
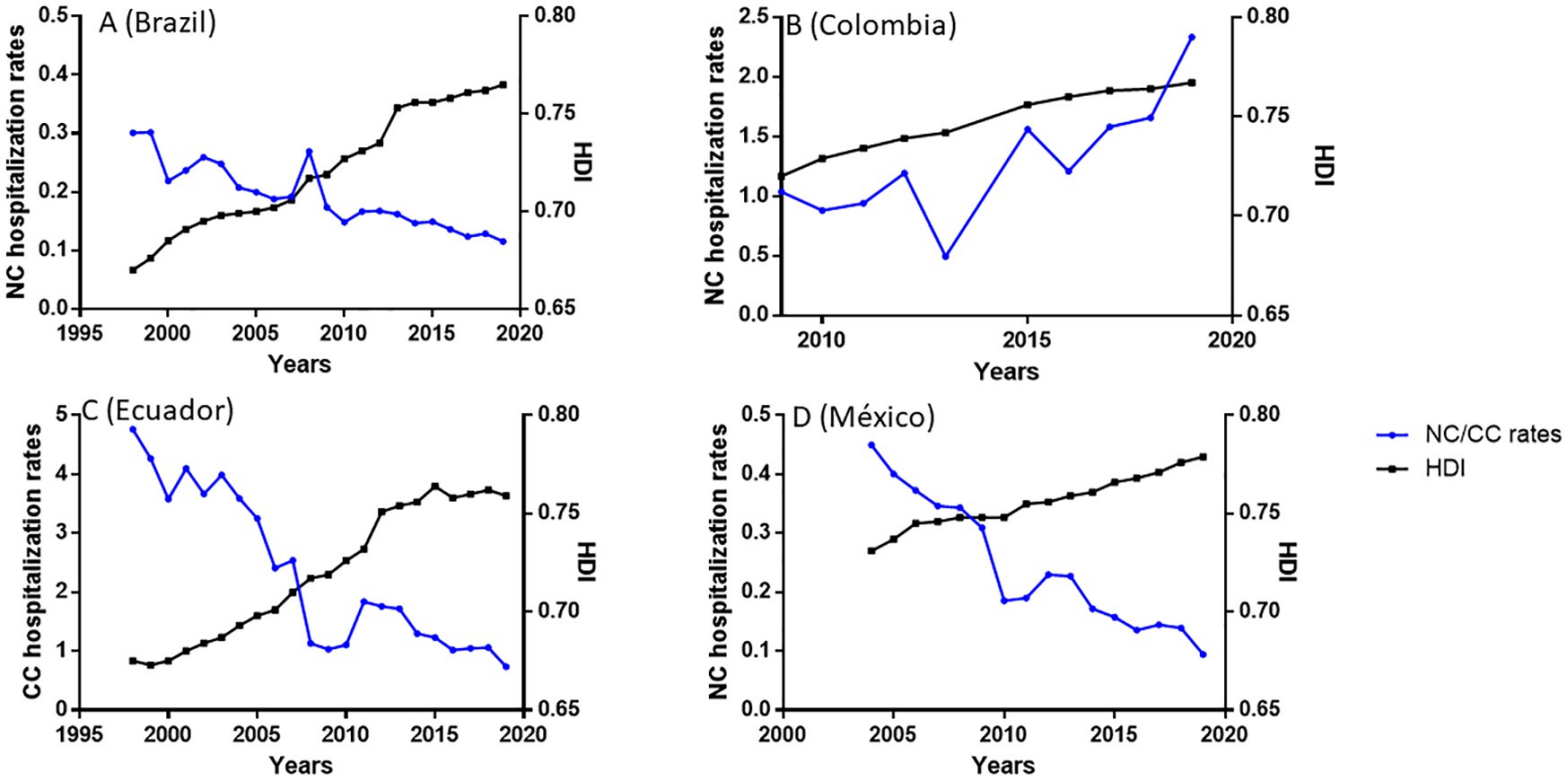
Evolution of the rates (per 100,000), Number of inpatients/Total population of the country. **A**: Brazilian National Public Health System (B69.0, SIH-SUS, 1998–2019). The evolution of the Human Development Index (HDI) over the same period in each country is also shown. **B**: NC cases registered in Colombia (B69.0, RIPS, 2009–2019). The evolution of the Human Development Index (HDI) over the same period in each country is also shown. **C**: Ecuadorian institutions of secondary level (B69, INEC, 1998–2019). The evolution of the Human Development Index (HDI) over the same period in each country is also shown. **D**: Mexican public institutions of secondary level (B69.0, DGIS, 2004–2019). The evolution of the Human Development Index (HDI) over the same period in each country is also shown.

**Fig 2:**
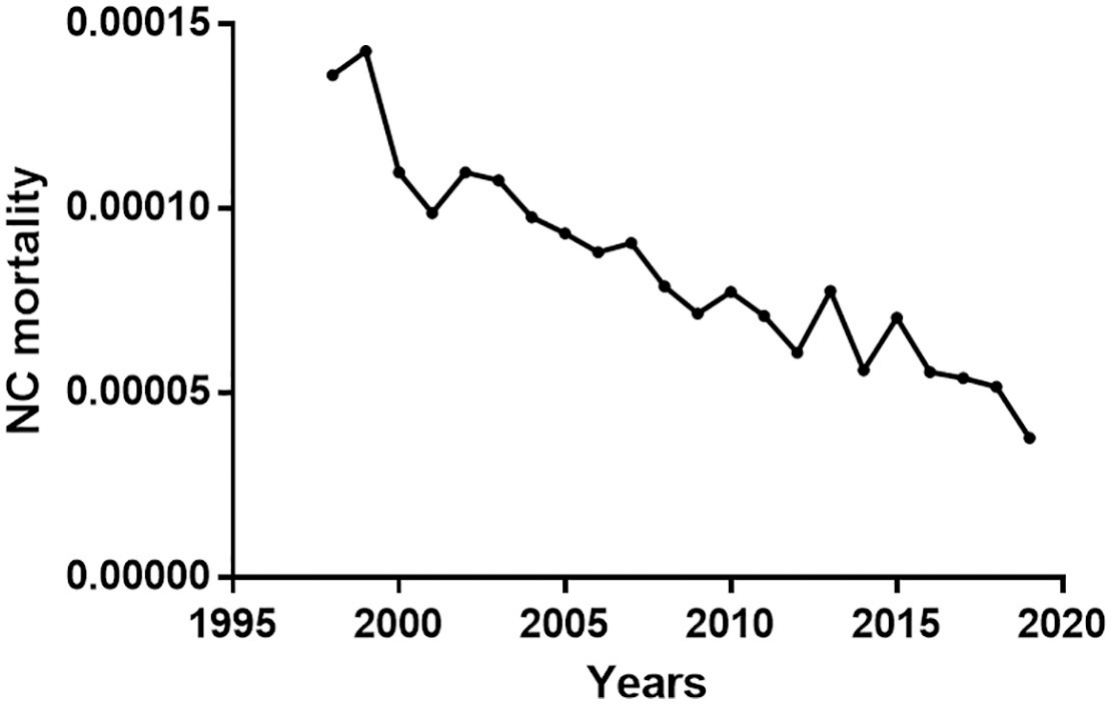
Evolution (1998–2019) of the rate, Number of deaths due to NC/Total number of deaths, in Brazil.

Over the study period, NC hospitalization and NC mortality rates significantly decreased (r = -0.931, p<0.0001; r = -0.886, p<0.0001, respectively, Figs 1A and 2). The highest NC hospitalization rate was in 1999 (0.30/100,000 population) and the lowest in 2019 (0.11/100,000 population). HDI significantly increased over the period (r = 0.999, p<0.0001), from 0.670 in 1998 to 0.765 in 2019, and a significant negative correlation between HDI and NC hospitalization rates appeared (r = -0.933; p<0.0001; Fig 1A). However, when adjusted for time, the association was no longer statistically significant (incidence rate ratio [IRR] per 0.01 HDI unit: 1.02, 95% confidence interval [CI]: 0.92–1.12, p = 0.709; Table 1).

**Table 1:** Association of Human Development Index with (neuro)cysticercosis hospitalizations by country, unadjusted and adjusted for time (calendar year)*.

Country (years)	Unadjusted for calendar year			Adjusted for calendar year		
	Incidence rate ratio†	95% CI	P-value	Incidence rate ratio†	95% CI	P-value
Brazil (1998–2019, n = 22)	0.92	0.90–0.93	<0.001	1.02	0.92–1.12	0.709
Colombia (2009–2019, n = 10)	1.19	1.07–1.31	0.002	0.83	0.46–1.52	0.556
Ecuador (1998–2019, n = 22)	0.86	0.83–0.86	<0.001	1.05	0.92–1.21	0.466
Mexico (2004–2019, n = 16)	0.67	0.60–0.75	<0.001	0.62	0.34–1.15	0.129

CI, confidence interval; HDI, Human Development Index.

*Negative binomial regression models were conducted for each country, offset by log (country population for each year).

^**†**^Per 0.01 unit change in HDI.

### Colombia

Over the study period, the NC hospitalization rate increased significantly (r = 0.83, p = 0.0047, Fig 1B), with the lowest in 2003 (0.49/100,000 population) and the highest in 2019 (2.33/100,000 population). HDI also increased significantly over the same period (r = 1, p<0.0001), from 0.720 in 2009 to 0.767 in 2019. A significant positive correlation between NC rate and HDI (r = 0.83, p = 0.005) emerged. However, when adjusted for time, the association was no longer statistically significant (IRR per 0.01 HDI unit: 0.83, 95% CI: 0.46–1.52; p = 0.556; Table 1).

### Ecuador

The evolution of hospitalization rates for cysticercosis between 1998 and 2019 in 39 secondary level institutions around the country (both public and private) are presented in Fig 1C. A clear and significant decrease in incidence is observed (r = -0.893, p<0.0001), which was highest in 1998 (4.76/100,000 population) and lowest in 2019 (0.73/100,000 population). On the other hand, over the same period, the HDI increased significantly (r = 0.983, p<0.0001), being at its lowest in 1999 (0.673) and highest in 2015 (0.764), and a significant negative correlation between HDI and cysticercosis hospitalization rates was observed (r = -0.859, p<0.0001). However, when adjusted for time, the association was no longer statistically significant (IRR per 0.01 HDI unit: 1.05, 95% CI: 0.92–1.21; p = 0.466; Table 1).

### Mexico

**Fig 3:**
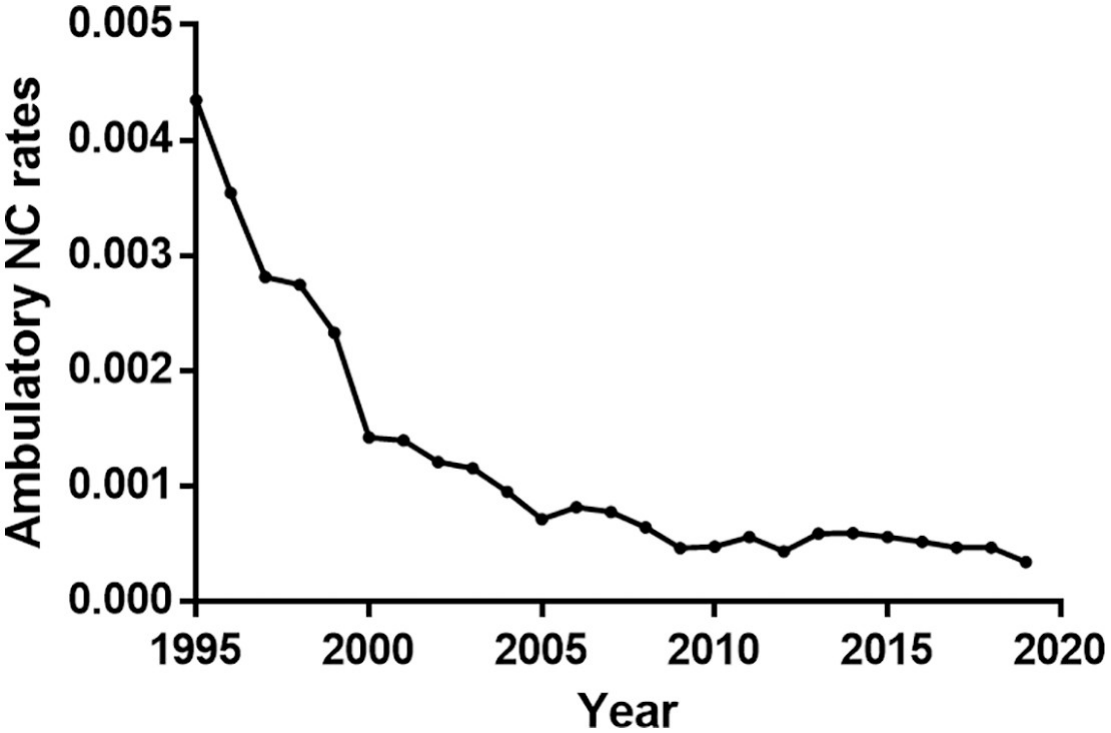
Evolution (1995–2019) of the rate, Number of outpatient consultations NC/Number of total outpatient consultations, in Mexican public institutions of primary level. (SUIVE).

Hospital discharges of patients with NC in all the Mexican secondary level public institutions showed a significant decrease between 2004 and 2019 (r = -0.964, p<0.0001; Fig 1D). The increase of HDI was also significant over the period (r = 0.997; p<0.0001), and a significant negative correlation between HDI and NC rates was observed (r = -0.953; p<0.0001; Fig 1D). However, when adjusted for time, the association was no longer statistically significant (IRR per 0.01 HDI unit: 0.62, 95% CI: 0.34–1.15, p = 0.129; Table 1). The same trend was observed with NC cases reported from ambulatory primary level clinics using the SUIVE data (r = -0.929; p = 0.0001; Fig 3).

**Fig 4:**
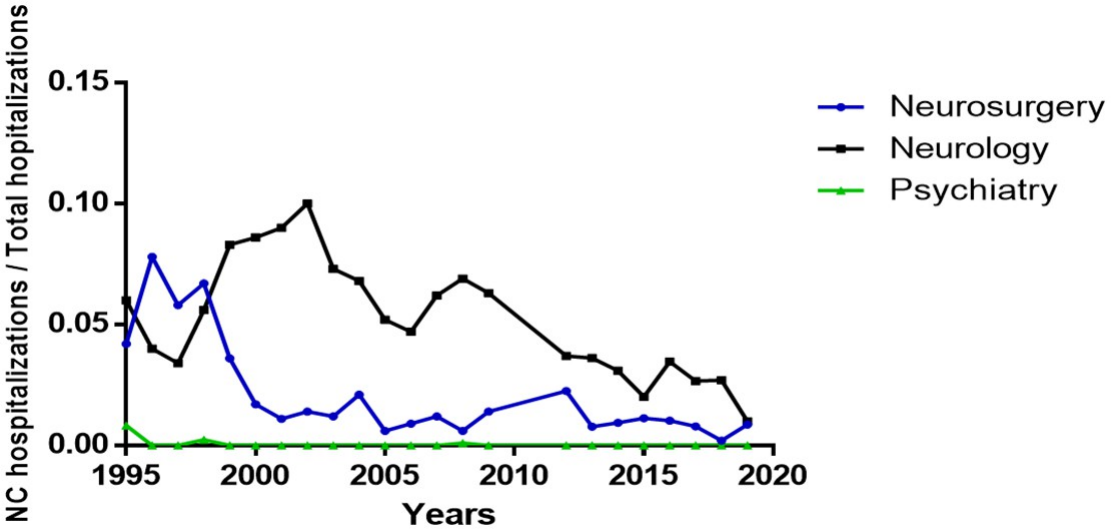
Rate of NC hospitalizations/Total hospitalizations in the departments of neurosurgery, neurology, and psychiatry at the INNN (Mexico City).

Fig 4 shows the admission rate per service at INNN during the 1995–2019 period. In neurosurgery, the admission rate for patients with NC was the highest in 1996 (7.8%) and the lowest in 2018 (0.21%), and a significant decrease was observed during the study period (r = -0.71; p = 0.001). Records of the neurology service also showed a significant decrease in the number of NC admissions in the last 25 years (r = -0.68; p = 0.003), with an annual decrease rate of 0.24%. The psychiatry service registered 5 individuals with NC, all of them before 2009.

**Fig 5:**
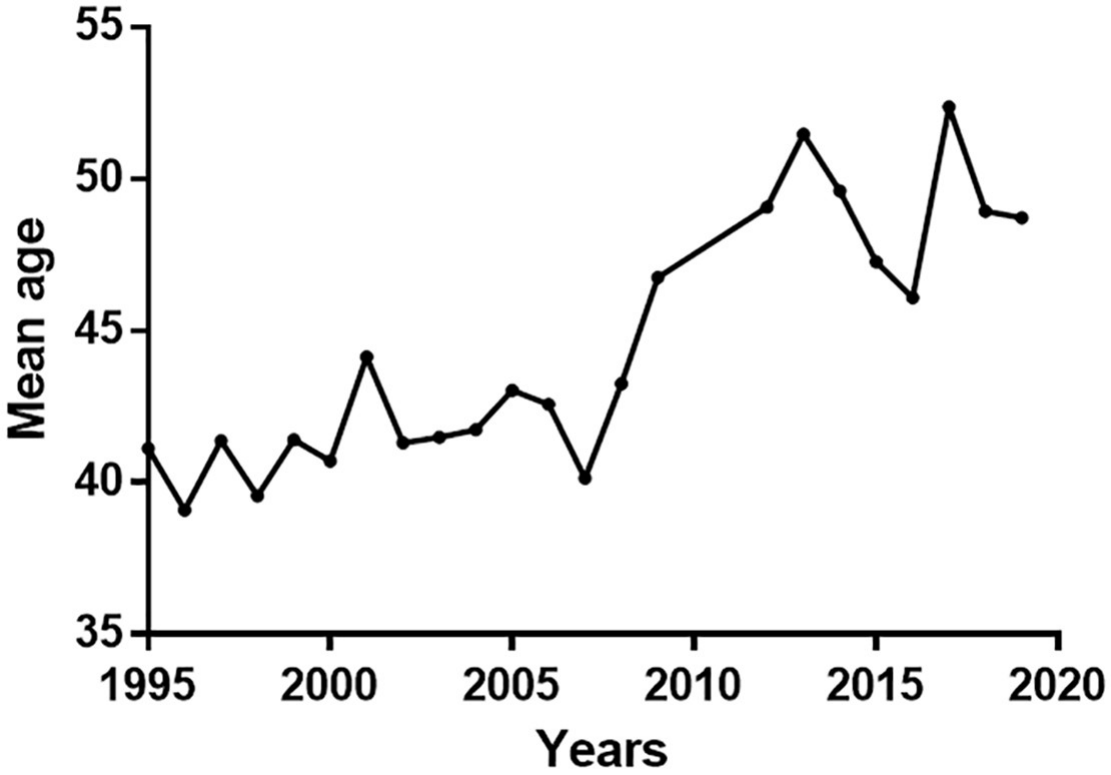
Mean Age of hospitalised patients with NC at the INNN (Mexico City) from 1995 to 2019.

The average age of patients hospitalised with NC between 1995 and 2019 was 44.45±4.02 (17–87 years), and a significant increase in patient age was observed during the study period (r = 0.839, p< 0.0001) (Fig 5). Thirty-two deaths were identified in patients hospitalised with NC from 1995 to 2019, with only four deaths in the last ten years and a mortality rate of 0.19/100,000 patients.

## Discussion

Data on the epidemiology of cysticercosis in endemic countries are often unavailable because most of them do not have official registries. Therefore, this study gathered information to assess the burden of cysticercosis in Latin American endemic countries using official records.

Data from Brazil, Ecuador, and Mexico are clear and significant. There are decreases in hospitalised cysticercosis rates (in the three countries), mortality rate (Brazil), and ambulatory attention (Mexico). Although in Ecuador, the available data consider all forms of cysticercosis (ICD code B69), data on cysticercosis and NC from Brazil and Mexico allow us to determine that NC accounts for approximately 90% of all recorded cysticercosis cases. Thus, the trend in cysticercosis also represents the trend of NC. Conversely, the trend regarding NC burden in Colombia increased significantly between 2009 and 2019.

The trends observed in Brazil, Ecuador, and Mexico are impressive and commendable. Since cysticercosis is clearly a marker of poverty in pork-eating countries, the decrease in NC hospitalization rate may be related to increasing HDI. In Mexico, this tendency was previously reported [10]. However, HDI was not significantly associated with NC hospitalization rate for any country when adjusting for time, suggesting that other time-related factors may explain changes in NC hospitalization incidence. For example, in Mexico, since 1994, specific measures aimed at preventing infection have been developed, a result of the awareness of health authorities from extensive and multidisciplinary research on the different facets of *Taenia solium* pathologies in the country [11]. Compulsory notification of NC was included, as it was in a pilot project in Ribeirao Preto, Brazil [12]. This measure has the major advantage to provide accurate quantification of the NC incidence and prevalence allowing the rational use of resources in control programs [13]. Different efficient methods are available for control programs (for example, treatment of taeniasis, treatment and vaccine of pigs, improvement of sanitary conditions, health education) [14], some of them at low costs, facilitating their implementation in resource-constrained countries [15].

Despite these encouraging trends, we must note that cases persist in these countries and attention to NC must continue. Furthermore, we must be aware that most of the data used are hospital-based and do not represent non-hospital settings, especially in the private sector. Further studies are needed to assess the NC burden in such settings.

The upward trend in Colombia is alarming, but various factors may be involved. Because the diagnosis of NC requires neuroimaging, increased availability of neuroimaging may give a false impression of an increase in incidence related to increased diagnosis. It is also possible that the RIPS data do not represent the overall NC situation in the country. Although RIPS data have been used in several studies allowing for the observation of trends of interest [6,16,17], it has also been argued that selection bias may occur because not all the population has access to the health services considered in this registry [16]. Some paradoxical trends occurred when comparing case notifications between the period 2009–2013 and the period 2015–2019; for example, an increase of more than 300% in the number of cases reported in 9 departments is observed (781 between 2009–2013 vs. 2,456 between 2015 and 2019), while in one department an abrupt decrease in registered cases occurs (from 171 to 19 cases). Whatever the factors involved in these trends, the data presented here should prompt authorities to evaluate them thoroughly and take appropriate action. In 2019, a control program for taeniasis/cysticercosis was proposed to be created, but it has yet to be implemented.

Although similar NC trends are observed in Brazil, Ecuador, and Mexico, it is also relevant to highlight the differences. Ecuador has a significantly higher cysticercosis burden than Brazil and Mexico (Fig 1). Although the cysticercosis rate in Ecuador is decreasing markedly, its rates in 2019 are still higher than the NC rates in Brazil and Mexico at the beginning of the period considered in this study. It is possible that this difference is linked to the current persistence of a higher proportion of the rural population in Ecuador (about 35%). In contrast, less than 20% of the population lives in rural areas in Brazil and Mexico (https://www.indexmundi.com/es/datos/indicadores/SP.RUR.TOTL.ZS/compare?country=co#country=br:ec:mx).

There is currently no marker to assess the endemic status of a country. However, it has recently been proposed that the decrease in the ratio of parenchymal to extraparenchymal NC and the reduction in ocular cysticercosis cases may be related to a decrease in infection pressure. Ocular cysticercosis is usually diagnosed soon after infection, parenchymal NC about five years after infection, whereas extraparenchymal NC can be diagnosed up to 20 years after infection. Thus, in a situation of decreased infection pressure, ocular cysticercosis will disappear first, and the parenchymal/extraparenchymal NC ratio will decrease [18]. There have been no reports of ocular cysticercosis cases in Mexico at DGIS for almost two decades. In Brazil, only 3 cases of ocular cysticercosis have been recorded at SIH-SUS since 2009, supporting the reduction in the parasite load and rate of new infections in these countries. Conversely, during the same period, 361 cases of ocular cysticercosis were registered at the RIPS health information system in Colombia. These data confirm the trends in NC rates. Moreover, given the differences in population size (approximately 210 million in Brazil, approximately 126 million in Mexico, and approximately 51 million in Colombia), these trends indicate the persistence of very active transmission in Colombia.

Another exciting observation is the steady increase in the age of hospitalised patients with NC in a tertiary level attention hospital (INNN) in Mexico (Fig 5), which could also be linked to a reduction in the number of recent infections. Most patients recently diagnosed in Mexico could have been infected decades ago, when the infection pressure was higher, explaining the increase in the age of hospitalised patients. Between 1995 and 2001, hospitalised patients with NC at INNN were predominantly in the 31–40 age group [19]. Between 1994 and 2004 in the same institution, the average age of patients with NC, hospitalised and ambulatory, was 38 to 39 years [8]. Between 2000 and 2014, the average age of patients seen in the INNN NC department was 41–42 years for patients with an extra-parenchymal form and 38 years for patients with a parenchymal form [3]. Our current results show that since 2007, the average age of over 46 years reflects a change in the demographic characteristics of the patients, compatible with a decrease in recent infections. These results remain solid considering the evolution of the average age of the Mexican population. Even though the average age has risen from 21 years in 1995 to 29 years in 2020 [20], 60% of the Mexican population is still under 20.

This study has some limitations to be considered. We worked with secondary data and there exists the possibility of coding errors. Indeed, it has been shown that the use of ICD coding is not harmonized between countries, nor within the same country, which could have affected our results [21]. Although notification of the diagnosis of hospitalised patients to the health authorities is mandatory, it is possible that some hospitals, especially in the private sector, do not always notify health authorities about their cases. Also, the diagnostic criteria considered by the national registries depend on the medical team that attend the patients and could perhaps lead to misdiagnosis in some cases. Data validation to avoid misclassification bias should be an important measure to consider in the future. However, it is likely that if there is a bias, it will be consistent throughout the study period, not affecting the trends in incidence. Notwithstanding, these data, the only ones available, represent a significant effort on the part of the health authorities, and we do believe that they are important to take in account.

## Conclusions

While it is clear that people with NC are still diagnosed in the endemic countries included in this study, the data provided here show a reduction in the burden of NC in Brazil, Ecuador, and Mexico. At the same time, an increase is noted in Colombia. Unfortunately, our study could only include 4 countries in the region, and we have no information to evaluate the situation in the others. Indeed, there is an almost total lack of reliable information in some countries of the region, for example in Central America, but the persistence of rural conditions in these countries indicates the likely presence of an active problem. It is therefore necessary to pay special attention to NC in these countries.

Regarding the countries included in this report, although rigorous interpretation of these trends requires better assessment of the situation of outpatients treated in private and public facilities, the NC trends in Brazil, Ecuador and Mexico are good news, especially in the context of the PAHO/WHO action plan to eliminate neglected tropical diseases in the region [22]. The differences observed with Colombia are striking, and efforts to understand the factors involved and to provide solutions need to be made.

NC is a potentially eradicable disease and efforts to control and eliminate it must continue. The encouraging results presented here should motivate interdisciplinary and inter-institutional cooperation to meet this challenge, as they demonstrate that the likelihood of control is possible and seems closer than ever.

## Supporting information

S1 Data Crude data of the four included countries used for the elaboration of the manuscript. (XLSX)
